# Spin excitations in nanographene-based antiferromagnetic spin-1/2 Heisenberg chains

**DOI:** 10.1038/s41563-025-02166-1

**Published:** 2025-03-14

**Authors:** Chenxiao Zhao, Lin Yang, João C. G. Henriques, Mar Ferri-Cortés, Gonçalo Catarina, Carlo A. Pignedoli, Ji Ma, Xinliang Feng, Pascal Ruffieux, Joaquín Fernández-Rossier, Roman Fasel

**Affiliations:** 1 Empa—Swiss Federal Laboratories for Materials Science and Technology, Dübendorf, Switzerland; 2 Faculty of Chemistry and Food Chemistry, and Center for Advancing Electronics Dresden, Technical University of Dresden, Dresden, Germany; 3 Max Planck Institute of Microstructure Physics, Halle, Germany; 4 International Iberian Nanotechnology Laboratory, Braga, Portugal; 5 Universidade de Santiago de Compostela, Santiago de Compostela, Spain; 6 Departamento de Física Aplicada, Universidad de Alicante, San Vicente del Raspeig, Spain; 7 University of Chinese Academy of Sciences, Beijing, People’s Republic of China; 8 University of Bern, Bern, Switzerland

**Keywords:** Magnetic properties and materials, Quantum mechanics, Magnetic properties and materials

## Abstract

Antiferromagnetic Heisenberg chains exhibit two distinct types of excitation spectrum: gapped for integer-spin chains and gapless for half-integer-spin chains. However, in finite-length half-integer-spin chains, quantization induces a gap, requiring precise control over sufficiently long chains to study its evolution. Here we create length-controlled spin-1/2 Heisenberg chains by covalently linking Olympicenes—Olympic-ring-shaped magnetic nanographenes. With large exchange interactions, tunable lengths and negligible magnetic anisotropy, this system is ideal for investigating length-dependent spin excitations, probed via inelastic electron tunnelling spectroscopy. We observe a power-law decay of the lowest excitation energy with length *L*, following a 1/*L* dependence in the large-*L* regime, consistent with theory. For *L* = 50, a V-shaped excitation continuum confirms a gapless behaviour in the thermodynamic limit. Additionally, low-bias current maps reveal the standing wave of a single spinon in odd-numbered chains. Our findings provide evidence for the realization of a one-dimensional analogue of a gapless spin liquid within an artificial graphene lattice.

## Abstract (web summary)

Open-shell nanographenes are used to fabricate length-controlled antiferromagnetic spin-1/2 Heisenberg chains. It is revealed that the spin excitation spectra evolve from gapped to gapless following a power-law dependence on chain length, along with the visualization of the standing waves of confined single spinons.

## Main

In one-dimensional (1D) quantum magnets, spontaneous symmetry breaking is inhibited by strong quantum fluctuations^1^, leading to the emergence of quantum disordered many-body states such as the resonating-valence-bond states^2,3^. The spin-1/2 Heisenberg chain with antiferromagnetic (AF) coupling stands as a flagship model in quantum magnetism, described by the Hamiltonian

1 $$\hat{{\mathcal{H}}}=J\sum _{i}{\hat{{\mathbf{S}}}}_{i}\cdot {\hat{{\mathbf{S}}}}_{i+1},$$

where *J* > 0 implies an AF nearest-neighbour exchange coupling. Although the AF coupling guarantees the formation of singlet pairs, quantum fluctuations render these singlet pairs resonating between different configurations, resulting in a 1D analogue of the gapless spin liquid^4^. In such a system, spin correlations in the ground state decay inversely with spin–spin separation, reflecting how quantum fluctuations prevent the formation of Néel ordering^5^. In the thermodynamic limit, the Bethe ansatz provides the analytical expressions for the ground state and excitations^6^. The ground state is a macroscopic singlet entangling all spins in the chain, and the excited states form a gapless continuum in the excitation spectrum, indicative of bound states comprising at least two fractional spin-1/2 excitations with well-defined energy–momentum relation, known as spinons^7^. For finite-length chains with the periodic boundary condition (PBC), the Lieb–Schultz–Mattis (LSM) theorem sets an upper energy bound for the lowest spin excitation energy^8^:

2 $${E}_{1}(L)-{E}_{0}(L)\equiv {\varDelta }_{{\rm{LEE}}}\le J\frac{2{\uppi }^{2}{S}^{2}}{L},$$

which converges to zero in the thermodynamic limit (*L*→∞), where *L* is the spin-chain length.

Despite the theoretical appeal, the experimental realization of the isotropic spin-1/2 Heisenberg model faces notable challenges. In quasi-1D crystals such as KCuF_3_ (ref. ^9^), CuGeO_3_ (ref. ^10^), CuPzN (ref. ^11^), Yb_2_Pt_2_Pb (ref. ^12^) and Cs_4_CuSb_2_Cl_12_ (ref. ^13^), interchain coupling induces transitions to dimerized or magnetically ordered phases. Additionally, the lack of access to well-defined finite chains hampers the systematic studies of the evolution of spin excitations with chain length as well as of the distinct behaviours of even- and odd-numbered chains. Advances in nanotechnology have led to the creation of artificial spin chains, such as atomic chains on surfaces^14–16^, quantum dot arrays^17^, optically trapped cold atoms^18,19^ and organometallic chains^20,21^. However, the fabrication of long chains with isotropic exchange interactions exceeding the dipolar coupling is challenging in these platforms.

The π-electron magnetism in graphene-derived nanomaterials has garnered substantial interest due to the substantial and controllable exchange couplings reaching several tens of millielectronvolts^22–27^. By covalently assembling selected open-shell nanographenes on a Au(111) surface, the Haldane gap and fractional edge states have been observed in *S* = 1 Heisenberg chains^28^. Additionally, a controlled transition between even and odd Haldane phases has been demonstrated in *S* = 1/2 dimerized Heisenberg chains^29^. Importantly, in nanographene-based systems, the covalent connections preserve the many-body interactions of individual magnetic nanographenes, ensuring that the spin excitations remain well separated from the frontier Hubbard bands of the chain^27^. Furthermore, the low atomic mass of carbon ensures negligible magnetic anisotropy and spin–orbit coupling^30^. These attributes make nanographenes an ideal platform for realizing and studying highly entangled quantum spin systems, with potential applications in insulator-based AF spintronics^31^.

**Fig. 1:**
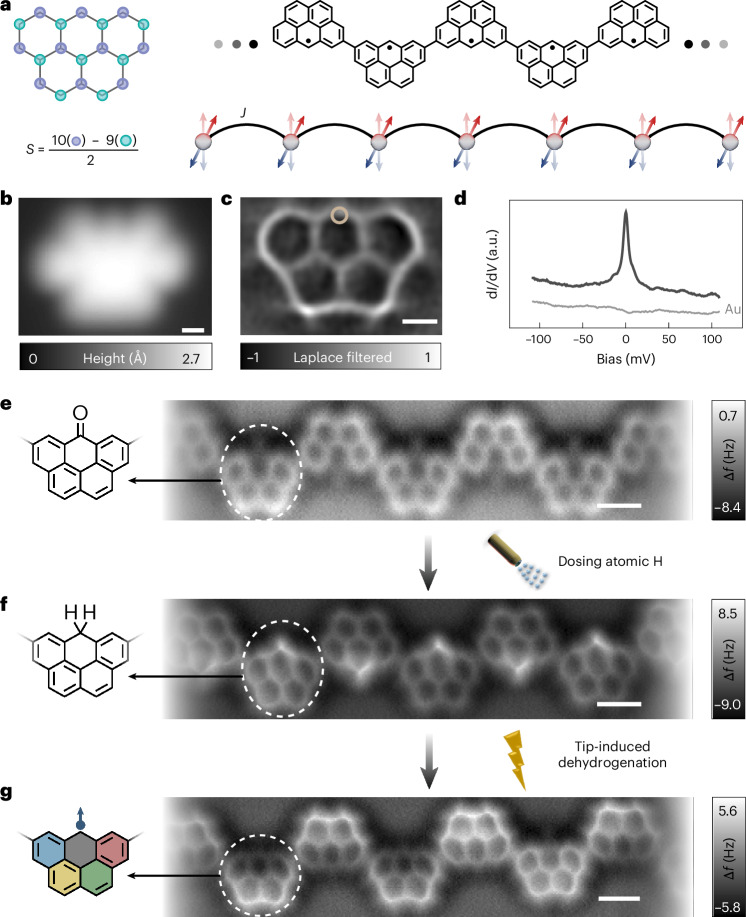
Fabrication of spin-1/2 Heisenberg chain using Olympicene. **a**, Structural illustration of a sublattice-resolved Olympicene monomer (left), and the chemical structure of the covalently linked Olympicene radical chain (right). The associated spin-chain model is illustrated below. **b**,**c**, STM image (*V*_bias_ = –50 mV, *I*_set_ = 500 pA; **b**) and Laplace-filtered nc-AFM image (**c**) of a single Olympicene. Scale bars (white), 0.5 nm. **d**, d*I*/d*V* spectra taken on an Olympicene monomer at the position marked by the brown circle in **c**, with the background spectrum taken on the Au(111) substrate shown by the grey curve. Acquisition parameters: *I*_set_ = 500 pA; lock-in root mean squared modulation voltage, *V*_mod_ = 1 mV. **e**–**g**, nc-AFM images of the Olympicene chain at various stages during synthesis: after polymerization (**e**), hydrogenation (**f**) and dehydrogenation (**g**). Repeat units are marked by the white dashed circles, with the corresponding structural models illustrated on the left.

Leveraging the potential of on-surface synthesis, we covalently connect specifically designed open-shell nanographenes, named Olympicenes^32^, into chains on a Au(111) surface to establish an isotropic spin-1/2 Heisenberg chain (Fig. 1a). According to the Ovchinnikov–Lieb rule^33–35^, each Olympicene has a spin-1/2 ground state due to a sublattice imbalance of one. We use scanning tunnelling microscopy (STM) and non-contact atomic force microscopy (nc-AFM) to characterize and manipulate the spin degrees of freedom along Olympicene chains. Spin excitations are probed using inelastic electron tunnelling spectroscopy^36–42^. Tip-induced dehydrogenation enables the precise manipulation of spin sites^26,43^, facilitating a systematic study of the evolution of spin excitations. Additionally, the ability to engineer a chain to have an odd number of units, whose ground state has *S* = 1/2, permits us to confine and probe a single spinon, circumventing the limitation of generating only paired spinons in excitation measurements^7,9,31,44,45^. These advantages enable the experimental observation of (1) a power-law decay of the lowest spin excitations with chain length *L*, showing a linear dependence on 1/*L* that falls below the LSM bound in the large-*L* region; (2) nearly ‘V-shaped’ d*I*/d*V* spectra for very long chains, which theory relates to the closing of the gap in the thermodynamic limit; and (3) standing waves of a single spinon in odd-numbered chains.

Figure 1b,c shows the structural characterization of an individual Olympicene molecule on a Au(111) surface using both STM and nc-AFM, respectively. The d*I*/d*V* spectrum taken on it displays the sharp Kondo peak (Fig. 1d), characteristic of a spin-1/2 unit on a metallic surface^23,43^. Apart from the low-energy spin degrees of freedom, the frontier orbitals of Olympicene are also characterized in Supplementary Note I. Covalently connecting Olympicenes through their majority carbon sites induces the nearest-neighbour AF exchange coupling *J* (Fig. 1a). This was experimentally achieved on Au(111) through the thermally induced polymerization of brominated precursor molecules (Fig. 1e), followed by hydrogenation, which replaces the C=O groups with CH_2_ groups^46^ and results in a hydrogen-passivated Olympicene chain (Fig. 1f). In the final step, spin sites were selectively activated by tip-induced dehydrogenation (Fig. 1g). Details of the sample preparation are available in the Methods and Supplementary Note II.

**Fig. 2:**
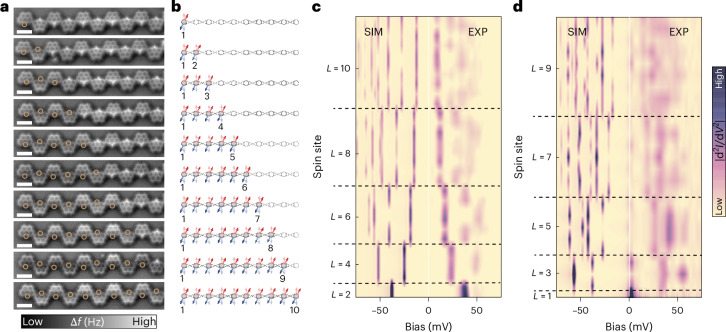
Spin excitations in short chains. **a**,**b**, nc-AFM images (**a**) and the corresponding schematics (**b**) of selectively activated spin chains. Effective length *L* from 1 to 10. Hydrogenated (passivated) Olympicene spin sites show bright protrusions in the nc-AFM image, whereas dehydrogenated (activated) spin sites do not. **c**,**d**, Spatially resolved colour map of the spin spectral weight (∣d^2^*I*/d*V*^2^∣) obtained at each spin site (brown circles) of chains with even (*L* = 2, 4, 6, 8 and 10; **c**) and odd (*L* = 1, 3, 5, 7 and 9; **d**) lengths. SIM, simulation; EXP, experiment. Higher intensities indicate greater probabilities of spin excitation. All spectra are acquired with *I*_set_ = 1 nA and *V*_mod_ = 1 mV. Simulated spin spectral weights using ED calculations are shown on the negative-bias side. Scale bars (white), 1 nm.

Figure 2a shows the step-by-step activation of a pre-passivated Olympicene chain, through which we obtained the spin chains of different effective lengths ranging from *L* = 1 to *L* = 10 (Fig. 2b). First, the nearest-neighbour exchange coupling *J* can be measured in a chain with an effective length of *L* = 2. The spin spectral weight, proportional to d^2^*I*/d*V*^2^ in experiments, reflects the energies of the excited states and their corresponding excitation probabilities. For *L* = 2, a single excitation occurs from the singlet ground state to the triplet excited state, with the energy difference indicating an exchange interaction of *J* ≈ 38 meV (Fig. 2c). This value is consistent with our multireference Hubbard model calculations for Olympicene dimers (Supplementary Note III).

Next, we investigate the length dependence of the low-lying spin excitations. The spin spectral weight for each spin site in different chains is shown in Fig. 2c,d, which displays a distinct odd–even behaviour around the zero bias: chains with an even number of units consistently show excitation gaps, with the gap size decreasing as *L* increases, whereas chains with an odd number of units display a zero-bias peak whose intensity decreases with increasing *L*. This is consistent with the fact that the ground state is an *S* = 0 singlet (*S* = 1/2 doublet) for even-numbered (odd-numbered) chains. It is worth noting that the zero-bias Kondo peak intensity for odd-numbered chains is notably weaker than that of a single spin 1/2. This reduction occurs because the spin 1/2 is highly delocalized across the entire chain, resulting in a much lower net spin density. The experimental observations align with the simulated spectral weight based on the exact diagonalization (ED) of the Hamiltonian in equation (1) with the experimentally determined *J* (Fig. 2c,d, negative-bias sides). We find that the excitations are associated with *S* = 1 (*S* = 1/2 and *S* = 3/2) states for even-numbered (odd-numbered) chains. Both theoretical calculations and experimental results show that for most excitations, the intensity of the d*I*/d*V* steps is modulated along the chain, reflecting the loss of translational invariance and the formation of standing waves. This modulation is missing in the case of rings, that is, chains with the PBC.

**Fig. 3:**
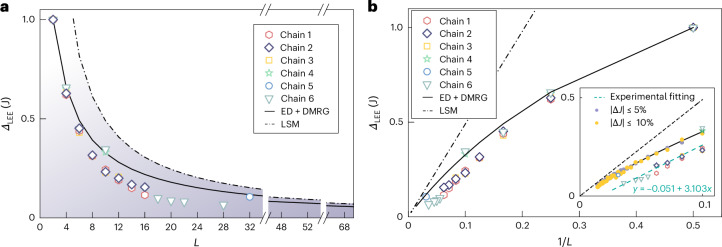
Evolution of *Δ*_LEE_ with *L.* **a**, Evolution of *Δ*_LEE_ with *L* (even-numbered chains only). Data from six different chains are shown (details in Supplementary Note IV). Results from ED and the theoretical value of the LSM bound are also shown as references. The region below the LSM bound is shaded for clarity. **b**, Relationship between *Δ*_LEE_ and 1/*L* (even-numbered chain lengths only). The inset shows the magnified view of the large-*L* region (1/*L* ≤ 0.1). A linear fit is applied, with the corresponding function displayed alongside the fitting line. Different random perturbations in *J* (Δ*J* within ±5% and ±10%) are considered in the DMRG calculations (Methods). Precision in determining the gap is ±2 meV, which is estimated based on the energy resolution of our measurement (~2 meV) and the broadening (~5 meV) of the excitation peaks in the d^2^*I*/d*V*^2^ spectra.

The evolution of the lowest excitation energies (*Δ*_LEE_) with *L* is monitored in six different chains (Fig. 3a, chains 1–6). The *Δ*_LEE_ value clearly decays with *L* in a power-law manner, in contrast to the exponential decay in gapped spin chains^28,29^. This is also confirmed by calculations obtained by using ED and density matrix renormalization group (DMRG) methods (Fig. 3a, solid black curve).

We note that the experimental data exhibit a slightly faster decay than theoretical predictions, suggesting a length-dependent renormalization of the excitation energy. We have considered potential factors such as Kondo screening from the metallic substrate^47–49^, longer-range exchange couplings and nonlinear exchange coupling terms. However, a quantitative agreement with this faster decay remains elusive, and we consider this an open question that warrants further investigation.

Nonetheless, the observed chain length dependence of *Δ*_LEE_ is consistent with the LSM theorem^8^. In the large-*L* region, *Δ*_LEE_ fits well with a linear relationship to 1/*L* (Fig. 3b (inset), green dashed line), lying below the LSM boundary. Although the LSM theorem is formulated for chains with the PBC, we demonstrate that *Δ*_LEE_ for the open boundary condition (OBC) chains of length *L* is always smaller than that for PBC chains of the same length (Supplementary Note V).

Furthermore, we have verified that the evolution of *Δ*_LEE_ towards zero is robust against perturbations in *J*. The colour-coded points around the DMRG curve (Fig. 3b, inset) show that random fluctuations in *J* of ±5% and ±10% have a negligible effect on the decay rate, thereby preserving the gapless character in the thermodynamic limit. In experiments, perturbations in *J* (less than ±10%) can arise from slight structural chain distortions induced by the Au(111) herringbone surface reconstruction and the mixing of inversion- and mirror-type connections along the chain (Supplementary Note VI), which accounts for slight variations observed between chains.

**Fig. 4:**
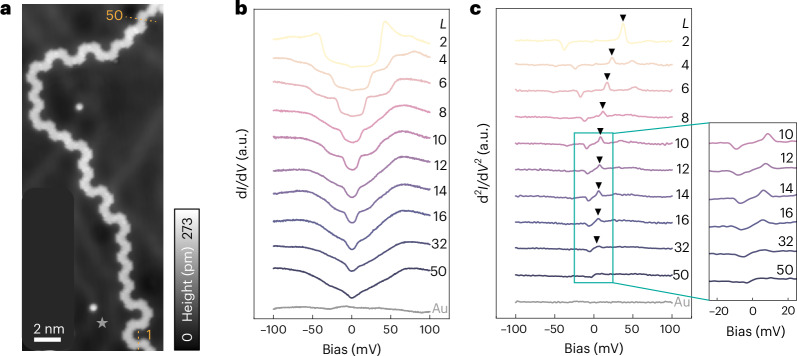
Spin excitation approaching the thermodynamic limit. **a**, STM image of an Olympicene chain with an effective length of *L* = 50. *V*_bias_ = –100 mV, *I*_set_ = 100 pA. The yellow dotted lines indicate the effective ends that are the boundaries between the activated and passivated spin sites. **b**, Inelastic electron tunnelling spectra taken from the middle region (averaged over sites *L*/2 and *L*/2 + 1) of chains with varying lengths. **c**, d^2^*I*/d*V*^2^ for chains of different lengths, obtained by numerically differentiating the spectra shown in **b**. Positions of *Δ*_LEE_ are indicated by the black triangles. A zoomed-in view for chains with *L* ≥ 10 is given. The background spectra taken on the Au(111) substrate are given by the grey curves, with the position marked out by a grey star in **a**. All the spectra are taken with *V*_mod_ = 1 mV and *I*_set_ = 1 nA.

For long chains in which the *Δ*_LEE_ value decays to a value comparable to the experimental energy resolution (~2 meV), the excitation spectra show a behaviour consistent with the thermodynamic limit. We examined the spin excitation spectra in a chain with an effective length of *L* = 50 (Fig. 4a), where we expect the spin excitation gap to be ~2 meV (based on Fig. 3a). In Fig. 4b, we compare the spin excitations measured at the centre of the chains (averaged over sites *L*/2 and *L*/2 + 1) with varying lengths. As *L* increases, the number of discernible excitation steps grows, evolving from single steps to multiple steps, and eventually to featureless slopes. For chains with lengths ranging from *L* = 12 to *L* = 32, only the lowest excitation steps are resolved, with their heights decreasing as *L* increases. All the densely packed higher excitation steps merge into a continuous slope. For *L* = 50, individual steps are no longer distinguishable within our energy resolution, and the entire spectrum transitions into a nearly V-shaped continuum, consistent with the behaviour expected near the thermodynamic limit. This evolution can also be monitored from the spin spectral weight (d^2^*I*/d*V*^2^), where the excitations are indicated by peaks/dips (Fig. 4c). The lowest-energy excitations decrease in both energy and intensity as *L* increases. For *L* = 50, no distinct excitation peaks are observed, highlighting the near-continuum character of the spectrum in the long-chain limit.

**Fig. 5:**
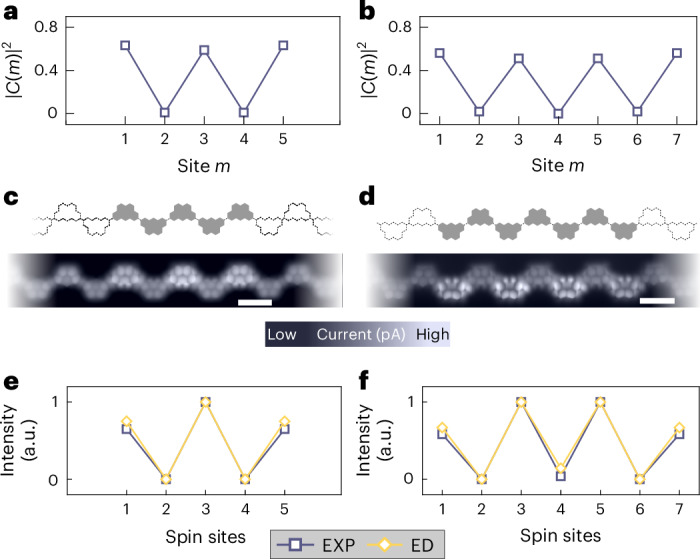
Imaging the standing wave of a single spinon. **a**,**b**, Theoretical calculation of ∣*C*(*m*)∣^2^, denoting the overlap of the ground state of the chains with *L* = 5 (**a**) and *L* = 7 (**b**) with localized spinons at site *m*. **c**,**d**, Schematic of the structures and low-energy (–5 meV) constant-height current images for Olympicene chains with five (**c**) and seven (**d**) effective units. The activated/passivated spin sites are denoted by grey/blank Olympicene geometries. **e**,**f**, Axial intensity profiles extracted from the current images in **c** (**e**) and **d** (**f**), symmetrized along the chain and normalized between 0 and 1. The zero-bias d*I*/d*V*, obtained from the ED calculations, are shown for comparison.

Finally, we focus on the odd-numbered chains that possess an *S* = 1/2 ground state, and demonstrate that it is the manifestation of a confined single spinon. As indicated by theoretical discussions regarding the nature of a single spinon^50^, the energy, momentum *q* and spin *S* = 1/2 of the spinons in PBC chains with length *L* = 2*n* (*n* = 1, 2, 3…) are well described through the construction of a linear combination of states in which an additional spin *↑* is inserted at site *m* (*m* = 1,…, 2*n* + 1) in the ground state of the spin chain (a similar reasoning can be applied when a spin *↓* is inserted instead). We label these states as |*Ψ*(*m*)〉 and refer to them as localized spinon states (Supplementary Note VII). Intriguingly, the state |*Ψ*(*q*)〉 ∝ ∑_*m*_e^i*qm*^|*Ψ*(*m*)〉, defined in the *L* = 2*n* + 1 PBC chain, has the same wavevector *q*, excitation energy and spin as a spinon in the *L* = 2*n* PBC chains. Importantly, we have found that the ground state of the *L* = 2*n* + 1 OBC chain (|*Ψ*_0_〉) can be expressed as a linear combination of the localized spinon states as |*Ψ*_0_〉 = ∑_*m*=1,2*n*+1_*C*(*m*)|*Ψ*(*m*)〉. In contrast to the case of extended single-spinon states |*Ψ*(*q*)〉 in PBC chains, the coefficients *C*(*m*) peak in the odd-*m* sites along the OBC chains (Fig. 5a,b). In other words, the ground state of the *L* = 2*n* + 1 OBC chain can be interpreted as a single-spinon standing wave (Supplementary Note VII) that has a strong modulation in local magnetization 〈*Ψ*_0_∣*S*_*z*_(*m*)∣*Ψ*_0_〉, which also peaks in the odd-numbered sites. Therefore, the single-spinon standing wave should result in a modulation of the amplitude of the Kondo peak along the chain. In the conventional theory for inelastic electron tunnelling spectroscopy^41,42^, the zero-bias conductance is related to the square of the local magnetization of the ground state, ∣〈*Ψ*_0_∣*S*_*z*_(*m*)∣*Ψ*_0_〉∣^2^, through resonant tunnelling via the Kondo effect.

To test this prediction, we have carried out low-bias current mapping in odd-numbered chains. As shown in Fig. 5c,d, the low-bias (–5 mV) current maps for *L* = 5 and *L* = 7 chains reveal strong modulation along the chains, with pronounced intensity at the odd-numbered active spin sites. Summing the intensity corresponding to each spin site provides a clearer view of the modulation along the chain axis (Fig. 5e,f), which exhibits a periodicity of two. This result is well reproduced by the simulated Kondo peak intensity from the ED calculations (ED data are shown in Fig. 5e,f) and also consistent with the single-spinon standing-wave picture outlined above (Fig. 5a,b).

We have successfully fabricated and characterized length-controlled nanographene chains realizing the gapless AF spin-1/2 Heisenberg quantum spin model. Our inelastic electron tunnelling spectroscopy results have revealed the lowest-energy spin excitations that decay linearly with 1/*L* and a V-shaped excitation continuum for long chains, evidencing a vanishing spin excitation gap in the thermodynamic limit, in line with the quasi-long-range spin–spin correlations that decay in a power-law rate. Importantly, the capability of building odd-numbered spin chains has allowed us to image a single-spinon standing wave. Given that spinons are quasiparticles that have largely evaded an intuitive picture, this is an important starting point for investigating spinon properties and interactions in prototypical quantum spin chains.

## Methods

### Sample preparation

Au(111) single-crystal surfaces were prepared by Ar^+^-ion sputtering followed by annealing at 430 °C. Precursor molecules (Supplementary Note II and VII) of Olympicene were deposited on a clean Au(111) surface, held at room temperature, via sublimation from a quartz crucible at ~150 °C. After molecule deposition, the sample was annealed at ~250 °C for 5 min to induce surface-assisted polymerization. Then, the sample was flashed to 300 °C several times to remove the detached Br atoms from the surface. Hydrogen reduction leading to the deoxygenation of the ketone groups was achieved by exposing the sample to atomic H produced by passing 3 × 10^−8^ mbar high-purity (99.999%) H_2_ gas for 10 h through a thermal gas cracker held at 1,500–1,600 °C.

### Spin manipulation methods

Tip-induced dehydrogenation is used to manipulate the spin sites. Initially, all the spins in the Olympicene chain are passivated following hydrogenation, due to the formation of -CH_2_- groups that pair with the originally unpaired π electrons. By applying a bias voltage of approximately 2.5 V using an STM tip, a single hydrogen atom can be selectively removed from the -CH_2_- group, transforming the *sp*^3^-configured -CH_2_- group into an *sp*^2^-configured -CH•- group, thereby contributing an unpaired π electron and activating the spin. Repeating this activation process on neighbouring molecules allows the creation of spin chains with perfectly controlled chain lengths.

### STM and scanning tunnelling spectroscopy measurements

STM and scanning tunnelling spectroscopy measurements were performed in a commercial low-temperature STM/nc-AFM system from Scienta Omicron operating at a temperature of 4.5 K and a base pressure below 2 × 10^−11^ mbar. All the d*I*/d*V* spectra and bond-resolved current images were acquired with a CO-functionalized tungsten tip. In situ cold deposition of CO molecules was performed to obtain a CO-functionalized tip. Differential conductance d*I*/d*V* spectra were acquired with a lock-in modulation frequency of 691 Hz, at an amplitude that is specified in the corresponding figure captions. The nc-AFM images were taken with a qPlus tuning fork sensor^51^ (resonance frequency, ~27 kHz; quality factor, ~27,000) with a CO-functionalized tungsten tip in the constant-height mode.

### CAS calculations

The starting point for the complete active space (CAS) calculations is a single-orbital tight-binding model that focuses exclusively on the *p*_*z*_ orbitals of the carbon atoms in the nanographenes. In our approximation, we consider both first- and third-nearest-neighbour hoppings, denoted as *t*_1_ and *t*_3_, respectively. The corresponding tight-binding Hamiltonian is given by

3 $${H}_{0}=-{t}_{1}\sum _{\sigma }\sum _{\langle i,\,j\rangle }{c}_{i,\sigma }^{\dagger }{c}_{j,\sigma }-{t}_{3}\sum _{\sigma }\sum _{\langle \langle \langle i,\,j\rangle \rangle \rangle }{c}_{i,\sigma }^{\dagger }{c}_{j,\sigma },$$

where ${c}_{i,\sigma }^{(\dagger )}$ denotes the annihilation (creation) operator of an electron with spin projection *σ* = *↑* and *↓* at site *i*. This single-particle model is diagonalized, resulting in a set of molecular orbitals.

At charge neutrality, an Olympicene chain with length *L* features *L* half-filled zero-energy states, which are slightly hybridized due to *t*_3_. For the CAS calculations, we consider a subset of molecular orbitals that includes these 2*N* zero-energy states, along with the two closest states in energy, to account for the Coulomb-driven exchange mechanism^48^. Thus, the active space comprises *N*_MO_ = *L* + 1 molecular orbitals. Then, we include interactions within the Hubbard model approximation, considering an on-site Hubbard repulsion *U*:

4 $${H}_{U}=U\sum _{i}{n}_{i,\uparrow }{n}_{i,\downarrow },$$

where ${n}_{i,\sigma }={c}_{i,\sigma }^{\dagger }{c}_{i,\sigma }$. The many-body Hubbard Hamiltonian *H*_0_ + *H*_*U*_ is represented in the restricted basis set, considering all the multielectronic configurations that can be obtained with *N*_e_ electrons in the *N*_MO_ molecular orbitals. Assuming half-filling, we always have *N*_e_ = *N*_MO_. The remaining electrons are, thus, assumed to fully occupy the molecular orbitals below the active space, implying that these are frozen, doubly occupied orbitals; the occupation of the molecular orbitals above the active space is also assumed to be frozen, featuring zero electrons. Finally, the resulting (truncated) Hubbard Hamiltonian is diagonalized numerically.

### DMRG calculations

DMRG calculations were carried out using the ITensor Julia library^52^. We used a protocol in which the maximum bond dimension is allowed to grow indefinitely as to keep the truncation error cut-off below 1 × 10^−8^, which ensures very high accuracy. We started the variational optimization with a randomized matrix-product state and stopped it when the energy and total spin were converged up to 1 × 10^−4^ meV and 1 × 10^−5^, respectively.

### Modelling d*I*/d*V* spectroscopy

To model the d*I*/d*V* spectroscopy, we consider a spin chain that is coupled to two electron reservoirs: the STM tip and the substrate. In our description, we consider two types of electron scattering that can produce a spin flip in a given site of the chain^53^: electrons that tunnel from the tip to the sample, exciting the spin chain in the process; and scattering between the substrate electrons. To compute the current *I*, we apply scattering theory including corrections up to the third order^42^; then, we differentiate the current with respect to the bias *V* (defined as the difference between the chemical potential of the two reservoirs), thereby obtaining the theoretical prediction for d*I*/d*V*. Up to the second order, the d*I*/d*V* spectrum is composed of thermally broadened excitation steps, whose height is determined by the spin spectral weight^41^. The spin spectral weight corresponding to a transition from a state *M* to a state *M*′ on a given spin site *i* is given by

5 $${{\mathcal{W}}}_{M,{M}^{{\prime} }}(i)={p}_{M}\sum _{a=x,y,z}| \langle {M}^{{\prime} }| {\hat{S}}_{a}(i)| M\,\rangle {| }^{2},$$

where *p*_*M*_ denotes the population of state *M*. This quantity reflects the probability of exciting state *M*′ (from state *M*, which is typically the ground state) with a local spin-flip interaction promoted by an STM tip placed on site *i*, with bias matching or exceeding the energy difference between *M* and *M*′. Excitations are only possible if the total spin *S* of the involved states respects the relation Δ*S* = 0, ±1. The third-order correction accounts for processes mediated by intermediate states; the spin conservation rule is enforced between the initial and final states, as well as between the initial/final state and the intermediate states. Energy conservation, however, is only required between the initial and final states. The third-order correction is responsible for the introduction of logarithmic resonances that lead to two main changes in the spectrum: (1) the thermally broadened steps acquire an overshooting feature at the onset of excitation; (2) a Kondo peak appears at zero bias if the system has a degenerate ground state. In our calculations, we have considered a temperature of 4.5 K.

The ED calculations were carried out using the QuSpin package^54^.

### Modelling influence of fluctuations in exchange

For the simulation of possible perturbations in the exchange coupling *J* along the Olympicene chain, to the reference value *J*, we added a random term δ*J*_*i*,*i*+1_ to the exchange between sites *i* and *i* + 1. The values of δ*J* were drawn from a uniform distribution over an interval of ±5%*J* and ±10%*J*. For every chain length, we computed the gap by averaging over at least 20 realizations of the disorder configuration.

## Online content

Any methods, additional references, Nature Portfolio reporting summaries, source data, extended data, supplementary information, acknowledgements, peer review information; details of author contributions and competing interests; and statements of data and code availability are available at 10.1038/s41563-025-02166-1.

## Supplementary information

Supplementary Information Supplementary Notes I–VIII and Figs. 1–15.

## Data Availability

The data that support the findings of this study are available via the Materials Cloud platform at 10.24435/materialscloud:zx-87 (ref. ^55^).

## Code availability

All relevant codes are available via the Materials Cloud platform at 10.24435/materialscloud:zx-87 (ref. ^55^).
