# Supplementary material for: Spin excitations in nanographene-based antiferromagnetic spin-1/2 Heisenberg chains
Source: Nat Mater. 2025 Mar 14;24(5):722–7. doi: 10.1038/s41563-025-02166-1 (PMC12048352; doi:10.1038/s41563-025-02166-1)
Supplement: Supplementary file 1 — Supplementary Notes I–VIII and Figs. 1–15. [file 41563_2025_2166_MOESM1_ESM.pdf]

nature materials

Supplementary information https://doi.org/10.1038/s41563-025-02166-1

Spin excitations in nanographene-based antiferromagnetic spin-1/2 Heisenberg chains

In the format provided by the authors and unedited

Contents:

Note I. Hubbard bands of olympicene chain

Note II. On-surface synthesis

Note III. CAS calculations for olympicene monomer and dimers

Note IV. Step-by-step activation along olympicene chains

Note V. Chains with OBC and PBC

Note VI. Perturbation in J

Note VII. Ground state of odd-chains as a single spinon state

Note VIII. Chemical synthesis of the precursor molecule

I. HUBBARD BANDS OF OLYMPICENE CHAIN

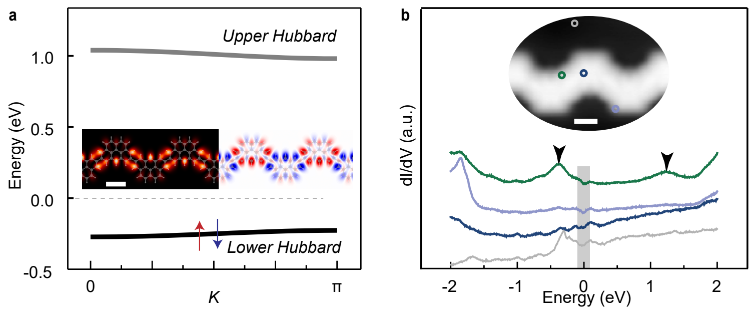

FIG. S1. **a**, DFT-calculated Hubbard bands of an infinite olympicene chain, with energy corrected by GW calculations for an olympicene dimer. The inset shows the calculated density of states distribution for the lower Hubbard band and the magnetization profile along the olympicene chain. **b**, Large energy scale dI/dV spectra taken on an olympicene chain, with positions indicated by color-coded circles in the inset STM images. The states corresponding to the upper and lower Hubbard bands are marked by black arrows. Parameters for dI/dV spectra: $I_{set} = 1$ nA, $V_{mod} = 10$ mV. STM image parameters: $V_{bias} = -100$ mV, $I_{set} = 100$ pA. The scale bar denotes 0.5 nm.

2

II. ON-SURFACE SYNTHESIS

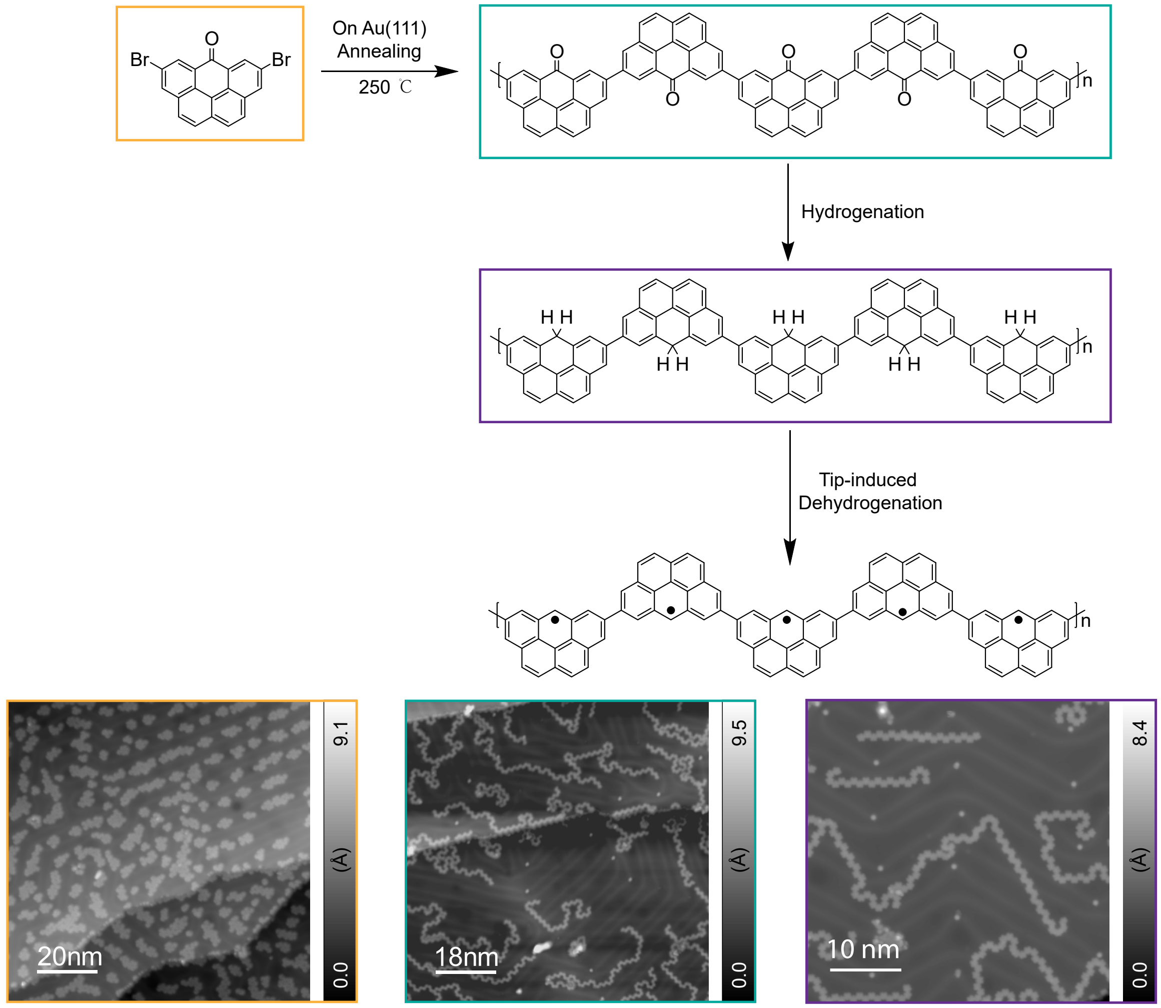

FIG. S2. On-surface reaction route, with the STM images for corresponding stages marke out by colored box. left: $V_{bias} = -600$ mV, $I_{set} = 100$ pA; middle: $V_{bias} = -500$ mV, $I_{set} = 500$ pA; right: $V_{bias} = -1500$ mV, $I_{set} = 50$ pA

III. CAS CALCULATIONS FOR OLYMPICENE MONOMER AND DIMERS.

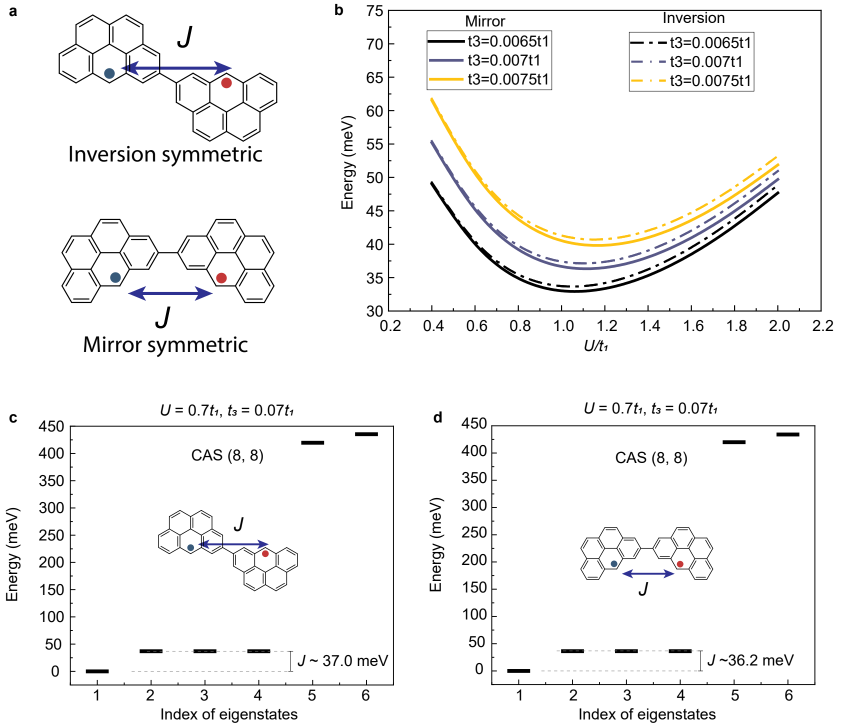

FIG. S3. **CAS calculation for examining exchange coupling J in olympicene dimers. a**, Structural illustration for olympicene dimers with inversion and mirror symmetries, respectively. **b**, Excitation energy from the singlet ground state to the triplet excited states of an olympicene dimer as a function of U/t_1, where U is the on-site Coulomb repulsion, $t_1 = 2.7$ eV is the hopping parameter for the nearest carbon sites and t_3 is for third-nearest neighbor carbon sites hopping. Both inversion and mirror type dimers are considered. The dependence on t_3 is considered, with different t_3s are used. The parameters that fit the experimental results are found to be $U/t_1 \sim 0.7$, and t_3=0.07 **c**, Eigenvalues of the ground state and the first several excited states for an inversion-type olympicene dimer using optimized parameters, with the lowest excitation energy indicated in the figure. **d**, Eigenvalues of the ground and the first several excited states for a mirror type olympicene dimer using the optimized parameters, with the lowest excitation energy indicated in the figure.

IV. STEP-BY-STEP ACTIVATION ALONG OLYMPICENE CHAINS

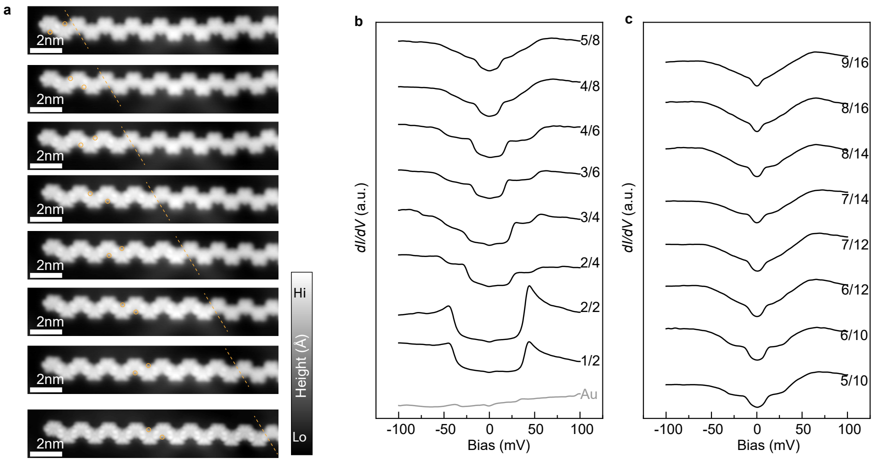

FIG. S4. **Data for chain-1**. **a**, STM images of the successively activated olympicene chain. The boundaries between activated and passivated spin sites are indicated by yellow dotted lines. STM image parameters: $V_{bias} = -1.5$ V, $I_{set} = 60$ pA. **b** and **c**, dI/dV spectra measured at the middle sites (L/2 and L/2+1) of the spin chain, with positions indicated by yellow circles in **a**. All spectra were recorded with $I_{set} = 1$ nA and $V_{mod} = 1$ mV.

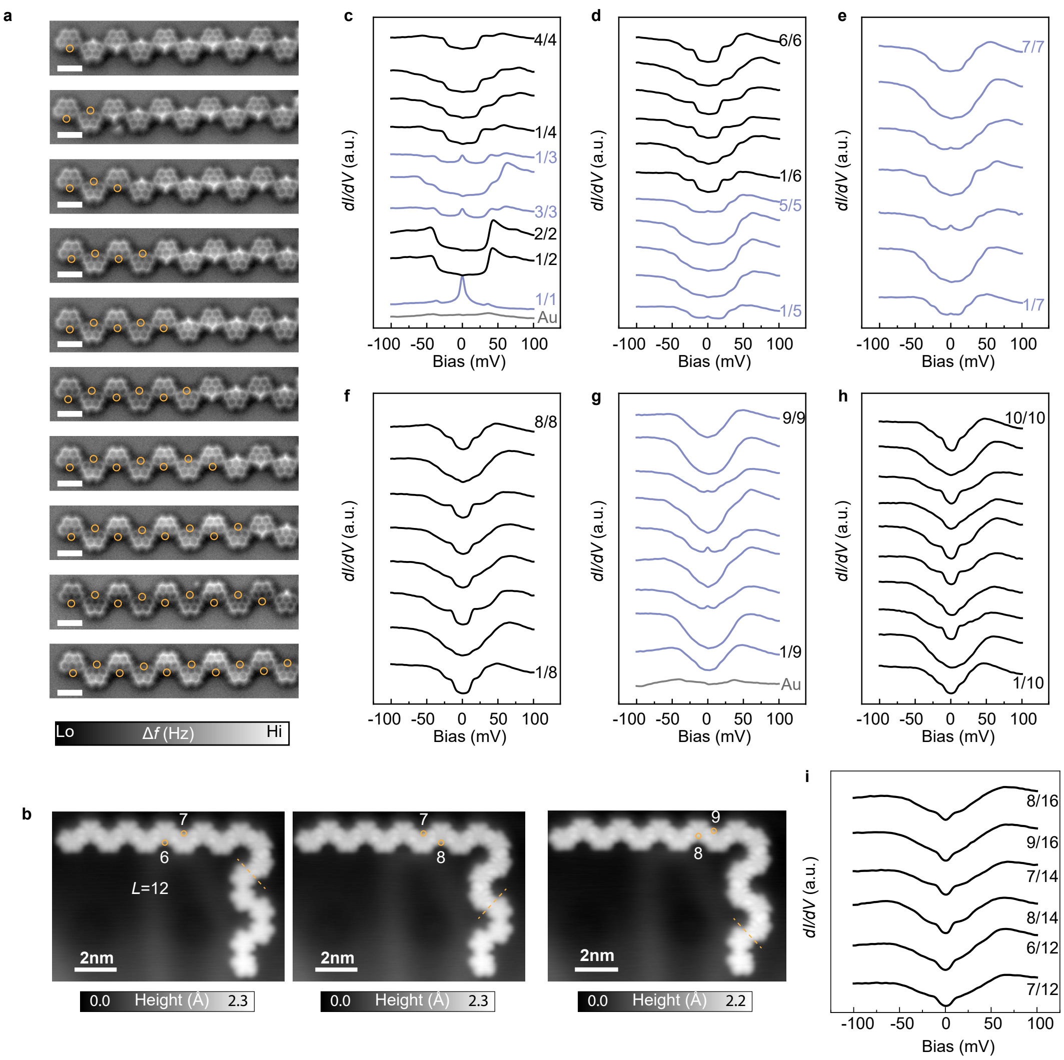

FIG. S5. **Data for chain-2, same chain with the one in Fig. 2 in the main text.** **a**, nc-AFM images of the successively activated olympicene chain, with effective lengths ranging from $L = 1$ to 10. White scale bars represent 1 nm. **b**, STM images of the successively activated olympicene chain (same as in **a**), with effective lengths from $L = 12$ to 16. STM parameters: $V_{bias} = -1.5$ V, $I_{set} = 100$ pA. **c**–**i**, dI/dV spectra measured at each spin site of the chain, with positions indicated by yellow circles in **a** and **b**. All spectra were recorded with $I_{set} = 1$ nA and $V_{mod} = 1$ mV.

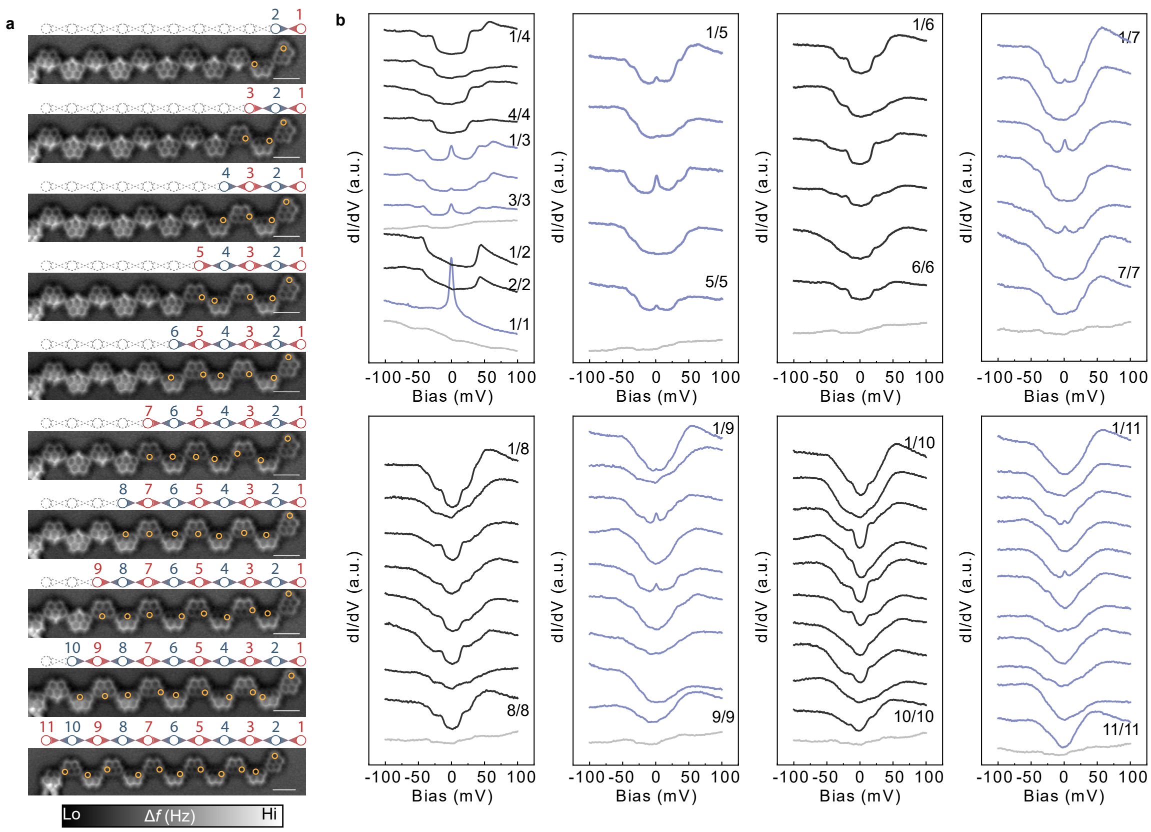

FIG. S6. **Data for chain-3**. **a**, nc-AFM images of the successively activated olympicene chain, with effective lengths ranging from $L = 1$ to 11. White scale bars represent 1 nm. **b**, dI/dV spectra measured at each spin site of the chain, with positions indicated by yellow circles in **a**. All spectra were recorded with $I_{set} = 500$ pA and $V_{mod} = 2$ mV.

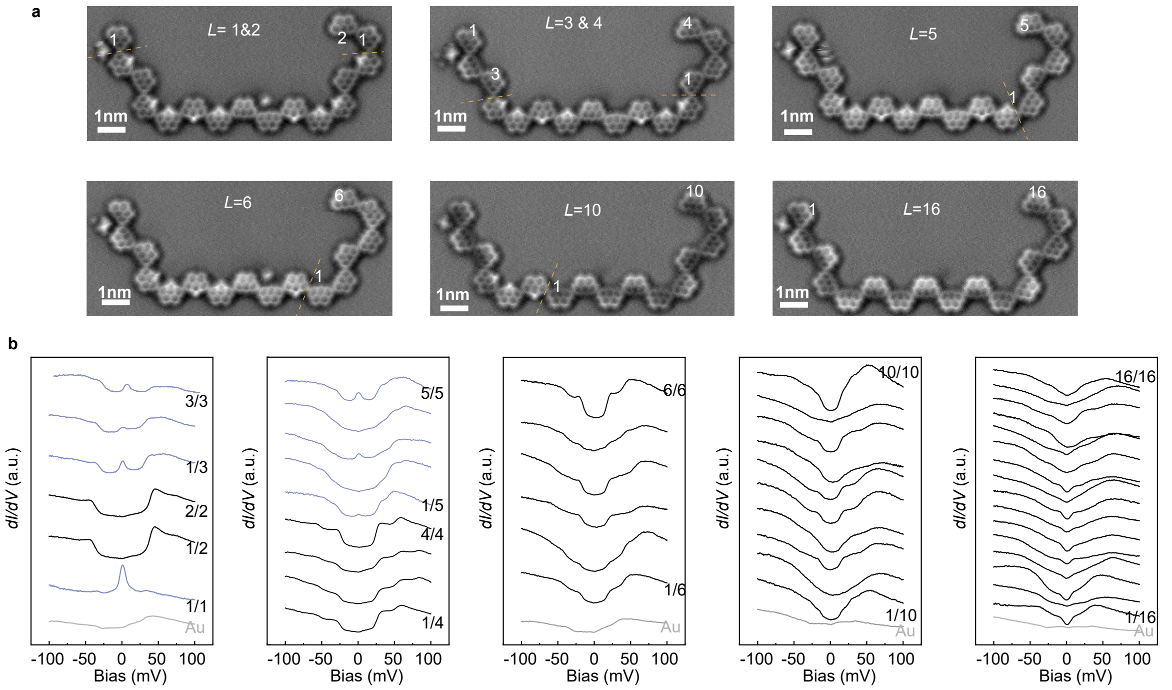

FIG. S7. **Data for chain-4**. **a**, nc-AFM images of the successively activated olympicene chain, with effective lengths of $L = 1$–6, 10, and 16. Yellow dotted lines indicate the boundaries between activated and passivated segments. The two ends of chains with different lengths are labeled with numbers (1 and L). **b**, dI/dV spectra measured at each spin site of the chain shown in **a**. All spectra were recorded with $I_{set} = 500$ pA and $V_{mod} = 2$ mV.

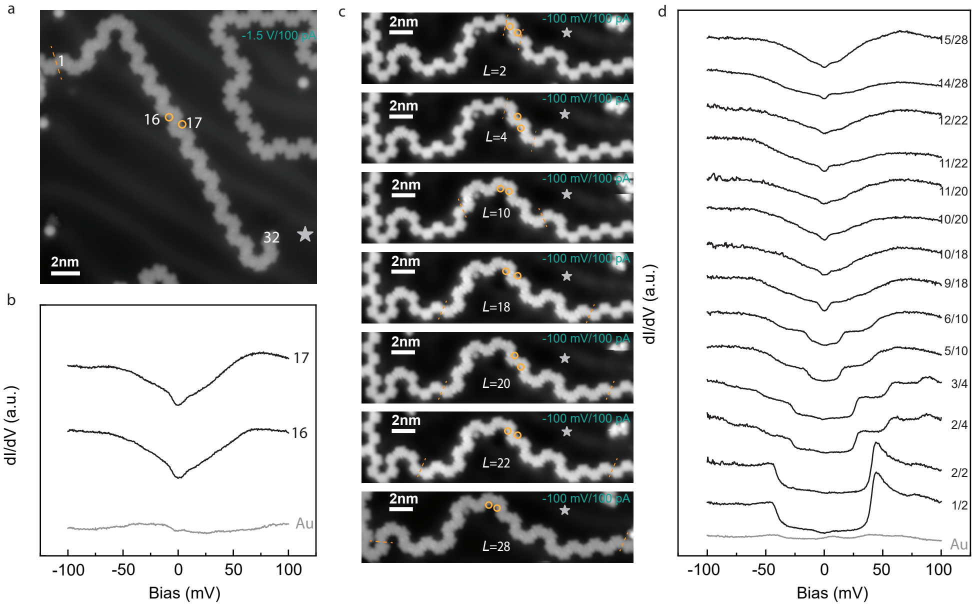

FIG. S8. **Data for chain-5 and chain-6.** **a**, STM images of an olympicene chain with an effective length of $L = 32$. Yellow dotted lines indicate the boundary between activated and passivated segments. The two ends and the middle sites of the chain are labeled with numbers. **b**, dI/dV spectra measured at middle sites of the chain shown in **a**, with positions indicated by yellow circles in **a**. The background spectra on Au are taken at the gray star position in **a**. **c**, successively activated olympicene chain, with effective lengths of $L = 2, 4, 10, 18, 20, 22, 28$. Yellow dotted lines indicate the boundaries between activated and passivated segments. **d**, dI/dV spectra measured at middle sites of chains shown in **c**, with the positions denoted by yellow circles. The background spectra on Au are taken at the gray star position in **c**. All spectra were recorded with $I_{set} = 1$ nA and $V_{mod} = 1$ mV.

V. CHAINS WITH OBC AND PBC

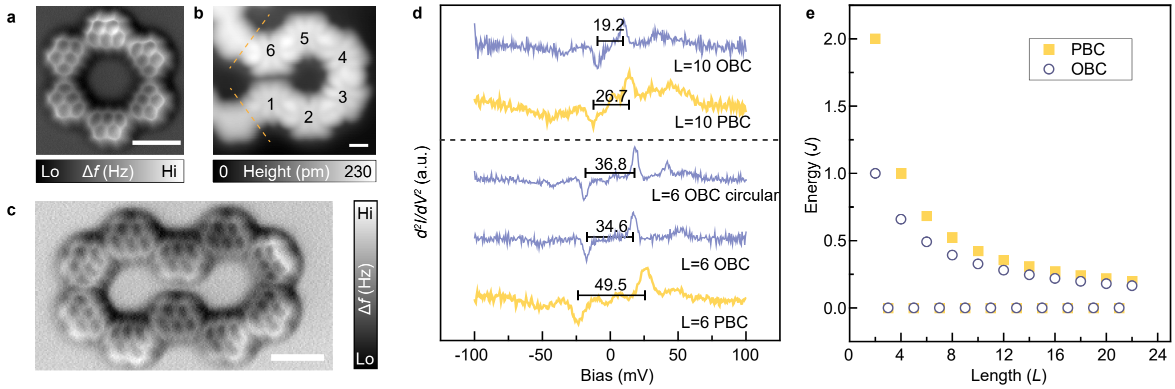

FIG. S9. **a** and **c**, nc-AFM images of $L = 6$ and $L = 10$ PBC rings. **b**, STM image of an $L = 6$ OBC chain with a circular geometry. ($I_{\text{set}} = 100$ pA, $V_{\text{bias}} = -100$ mV). The yellow dashed lines denote the effective chain ends. **d**, Comparison of d^2I/dV^2 between chains of the same length but different boundary conditions. Data for $L = 6$ and $L = 10$ OBC are taken from the chain-1 shown in Fig. S4. Data for $L = 6$ and $L = 10$ PBC are taken from the closed rings shown in **a** and **c**, respectively. Data for the $L = 6$ OBC circular is taken from the chain shown in **b**. White scale bars denote 1 nm. **e**, **e**, Comparison of ED-calculated Δ_{LEE} for chains with OBC and PBC at different lengths.

6 VI. PERTURBATION IN J

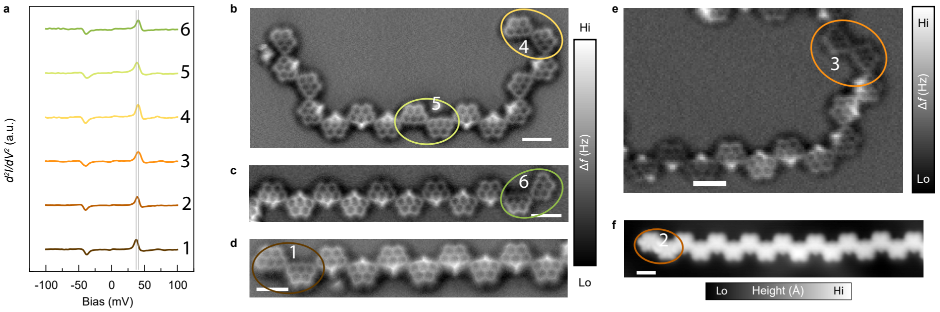

FIG. S10. **Perturbation in exchange coupling J.** **a**, Spin spectral weight (d^2I/dV^2) of different dimers within different chains. For dimers 1 and 2: $I_{set} = 1$ nA, $V_{mod} = 1$ mV; for dimers 3-6: $I_{set} = 500$ pA, $V_{mod} = 1$ mV; **b**, nc-AFM images indicating the positions of the dimers shown in **a**. White scale bars denote 1 nm.

VII. GROUND STATE OF ODD-CHAINS AS A SINGLE SPINON STATE

Here we relate the zero bias maps of dI/dV in odd-numbered chains with the magnetization of individual spinons. The rational is the following. Spinons are the $S = 1/2$ elementary excitations of Heisenberg spin chains. In even-numbered chains, they are always packed in pairs, quartets, etc, as the allowed spin values of the physical spin excitations are integer. However, it has been suggested[2] a connection between spinons in chains with N sites and quantum states build in $N + 1$ chains, using the following argument.

The starting point is the wave function of the ground state of a N chain.

$$|\Psi_0^{(L)}\rangle = \sum_j A_j^{(L)} |j\rangle \tag{1}$$

where $|j\rangle = |\sigma_1, ..., \sigma_L\rangle$ are the 2^L product states that can be defined with L spins. Since the ground state is eigenstate of the total S_z operator with null eigenvalue, the only non-vanishing coefficients $A_j^{(L)}$ are those for which $\sum_{i=1,L} \sigma_j = 0$.

Following the work of reference [2], we introduce the states $|j(m)\rangle$, that have one additional spin $\uparrow$, compared to $|j\rangle$ introduced at site m. Thus, for a given $|j\rangle$, with L entries, $|j(m)\rangle$ has $L + 1$ entries, where entries 1 to $m - 1$ are identical to j, entry m is $\uparrow$, and entries from $m + 1$ to $L + 1$ in $|j(m)\rangle$ are identical to entries m to L in j. States $|j\rangle$ are eigenstates of total $\hat{S}_z$, with eigenvalue S_z, then the corresponding $|j(m)\rangle$ are also eigenstates, with eigenvalue $S_z + \frac{1}{2}$.

Following the original work[2] of Kulka et al, we postulate the state

$$|\Psi^{(L+1)}(m)\rangle = \sum_{j(m)} A_j^{(L)} |j(m)\rangle \tag{2}$$

that live in a chain with $N + 1$ sites. These states are clearly singling out a special site, m in the $N + 1$ chain, and ensuring that its magnetization is going to be $+1/2$. These states also single out $S_z = +\frac{1}{2}$. Extension to the $S_z = -\frac{1}{2}$ is straightforward. For reasons that become clear below, we refer to these states as *localized spinon states*

In the original formulation[2], the chain has periodic boundary conditions, and it makes sense to introduce the states

$$|\Psi^{(L+1)}(q)\rangle = \frac{1}{\sqrt{L+1}} \sum_m e^{iqm} |\Psi^{(L+1)}(m)\rangle \tag{3}$$

where

$$q = \frac{2\pi n}{L+1} \tag{4}$$

with $-\frac{L}{2} \leq n \leq \frac{L}{2}$. Thus, q can take $L+1$ values, very much like m.

Importantly, it was shown[2] that excitation energy associated to these states in the $L+1$ chains

$$\epsilon_q \equiv \frac{\langle \Psi^{(L+1)}(q) | H_{L+1} | \Psi^{(L+1)}(q) \rangle}{\langle \Psi^{(L+1)}(q) | \Psi^{(L+1)}(q) \rangle} - E_G^{L+1} \tag{5}$$

is identical to the single spinon energy. The picture that emerges from this observation is that single spinons wave functions are completely defined by the ground state wave function coefficients of the chain with L, and amount to distribute an extra $\uparrow$ spin in a virtual $L+1$ chain. We note that the lowest excitation energy was obtained for q closest to $\pi/2$, i.e., for $n = \pm\frac{(L+1)}{4}$ Since $L+1$ is odd, there is no integer value of n that matches the condition, so, we can only approach the zero energy spinon, with $q = \frac{\pi}{2}$, in the thermodynamic limit.

We can summarize the picture we have just presented as follows:

1. States in Eq. (2), $|\Psi^{(L+1)}(m)\rangle$, introduce a $\uparrow$ "defect" in site m

2. Based on their match with the analytical formula for the energy dispersion of spinons, states in Eq. (3) are spinon states with well defined wave vector q. They have the same energy, momentum and spin.

3. Hence, we refer to $|\Psi^{(L+1)}(m)\rangle$ states, the ones that enter in the plane wave superposition defining $|\Psi^{(L+1)}(q)\rangle$, as "localized spinon" states.

We now ask ourselves the question: what is the relation between the ground state of the open-boundary condition with $L+1$ sites and the localized spinon states of Eq. (2)?. For that matter, we have computed the overlap of the ground state of the chain with $L+1$ sites, $|\Psi_0^{(L+1)}\rangle$, with the normalized localized spinon states:

$$C(m) \equiv \langle \Psi^{(L+1)}(m) | \Psi_0^{(L+1)} \rangle \tag{6}$$

We note that even though the m-spinon states are normalized, they are not mutually orthogonal. As a result, the sum over the $|C(m)|^2$ coefficients is not guaranteed to converge to unity. The results are shown in Fig. 5a and 5b of the main text. We find that the $|C(m)|^2$ coefficients are strongly peaked in the odd-sites of the chain. Therefore, we conclude that, very much like the single-spinon states with wave vector q in Eq. (3) are linear combinations of the localized spinons, with the same weight in all sites, the ground state of the odd-number

chain is also a linear combination of the localized spinon states, with a predominant weight on the odd-sites of the chain. Given that the localized spinon states $|\Psi^{(L+1)}(m)\rangle$ have a finite magnetization at site m, and given that our zero bias dI/dV feature a strong enhancement at odd sites, that theory relates to the $\langle\Psi_0^{(L+1)}|S_z(i)|\Psi_0^{(L+1)}\rangle$ profile, we conclude that our experiments are reflecting the formation of a single spinon wave packet.

In order to shed light on the nature of this wave-packet, we have computed the overlap of the ground state of $L+1$ chains with periodic boundary conditions with the localized spinon basis. It turns out that $L+1$ chains have a doubly degenerate $S = 1/2$ ground state. It is possible to assign to these two members of the doublet a quantum number associated to the C_{L+1} symmetry of the chain with PBC. The two doublets are eigenstates of C_{L+1} operator with eigenvalue $e^{\frac{\pm 2\pi i}{L+1}}$. We have computed the $C(m)$ coefficients of these ground states. They have the same moduli, constant across the chain, and an opposite winding phase. Hence, the two ground states of the chain with PBC are mathematically very similar to q-spinon states of Eq. (3). Now, we compute the overlap with the ground state of the chain with open boundary condition and we find an overlap of $f\frac{1}{\sqrt{2}}$, with f ranging from 0.97 to 0.92 for chains of length 4 to 18, respectively. We conclude that the ground state of the chain with open boundary condition is a standing wave formed as a linear combination of the two GS of the chain with PBC. In turn, these states are isomorphic to spinon states with well defined wave-vector. Therefore, the ground state of the chains with OBC is a single-spinon wave packet, and the resulting pattern of spinon density and the corresponding average magnetization cause the strong modulation of the zero bias Kondo peak along the chain.

VIII. CHEMICAL SYNTHESIS OF THE PRECURSOR MOLECULE

All the reagents were obtained from Sigma Aldrich, TCI, abcr, Strem and Chempur. All these chemicals were used as received without further purification. All reactions dealing with air- or moisture-sensitive compounds were carried out in a dry reaction vessel under an argon (Ar) atmosphere using standard vacuum-line and Schlenk techniques.

Thin layer chromatography (TLC) was performed on silica-coated aluminium sheets with a fluorescence indicator (TLC silica gel 60 F254, purchased from Merck KGaA).

Column chromatography was performed on silica (SiO_2, particle size 0.063-0.200 mm, purchased from VWR).

NMR spectra were recorded on a Bruker Avance III 600 spectrometer operating at 600 MHz for ^{1}H and 150 MHz for ^{13}C at 25°C. The NMR spectra were recorded using the standard Bruker pulse programs. $C_2D_2Cl_4$ ($\delta(^1\text{H}) = 5.98$ ppm, $\delta(^{13}\text{C}) = 73.7$ ppm)) was used as the solvent and as an internal chemical shift reference. Chemical shifts (δ) are reported in ppm. The following abbreviations are used to describe peak patterns as appropriate: d = doublet. The high-resolution matrix-assisted laser desorption/ionization time-of-flight (MALDI-TOF) mass spectrometry was performed on a Bruker Autoflex Speed MALDI TOF MS (Bruker Daltonics, Bremen, Germany) using dithranol as the matrix.

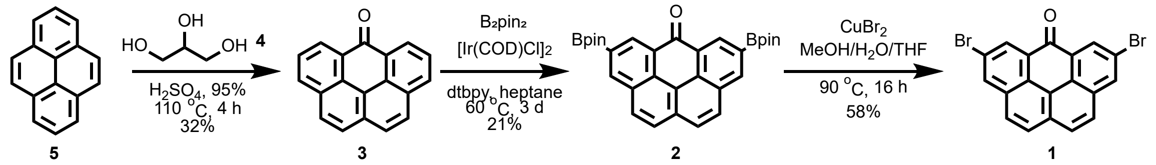

FIG. S11. Synthesis of the precursor 4,8-dibromo-6*H*-benzo[*cd*]pyren-6-one (**1**)

Compound **2** was synthesized as reported in the literature[1]. A 25 mL pressure tube was charged with compound **2** (110 mg, 0.217 mmol), copper (II) bromide (291.2 mg, 1.3 mmol), 2 mL tetrahydrofuran, 6 mL methanol and 6 mL water. The tube was degassed by argon bubbling for 10 min, then sealed and heated at 90 °C for 16 h. After cooling to room temperature, the mixture was extracted with dichloromethane three times (10 mL × 3). The combined organic layer was dried over magnesium sulfate, and then evaporated. The residue was purified by silica gel chromatography to yield the final product as a yellow solid (103.4 mg, 58%). Before submitting on-surface synthesis, **1** was recrystallized from

dichloromethane. ^{1}H NMR (600 MHz, $C_2D_2Cl_4$): δ 8.76 (d, 2.1 Hz, 2H), 8.40 (d, 2.1 Hz, 2H), 7.97 (d, 8.7 Hz, 2H), 7.95 (d, 8.7 Hz, 2H). ^{13}C NMR (150 MHz, $C_2D_2Cl_4$): δ 182.24, δ 136.99, 133.07, 132.44, 132.34, 130.84, 128.71, 128.29, 126.64, 121.76, 120.60. HR-MS MALDI-TOF (m/z): calculated for $C_{19}H_8Br_2O$ $[M]^+$, 409.8936; found, 409.8952 error = +3.9 ppm.

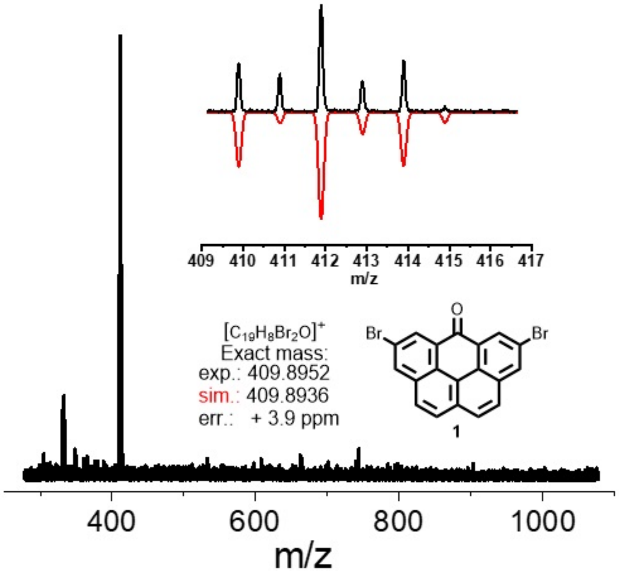

FIG. S12. HR MALDI-TOF mass spectrum of **1**: full spectrum and isotope pattern.

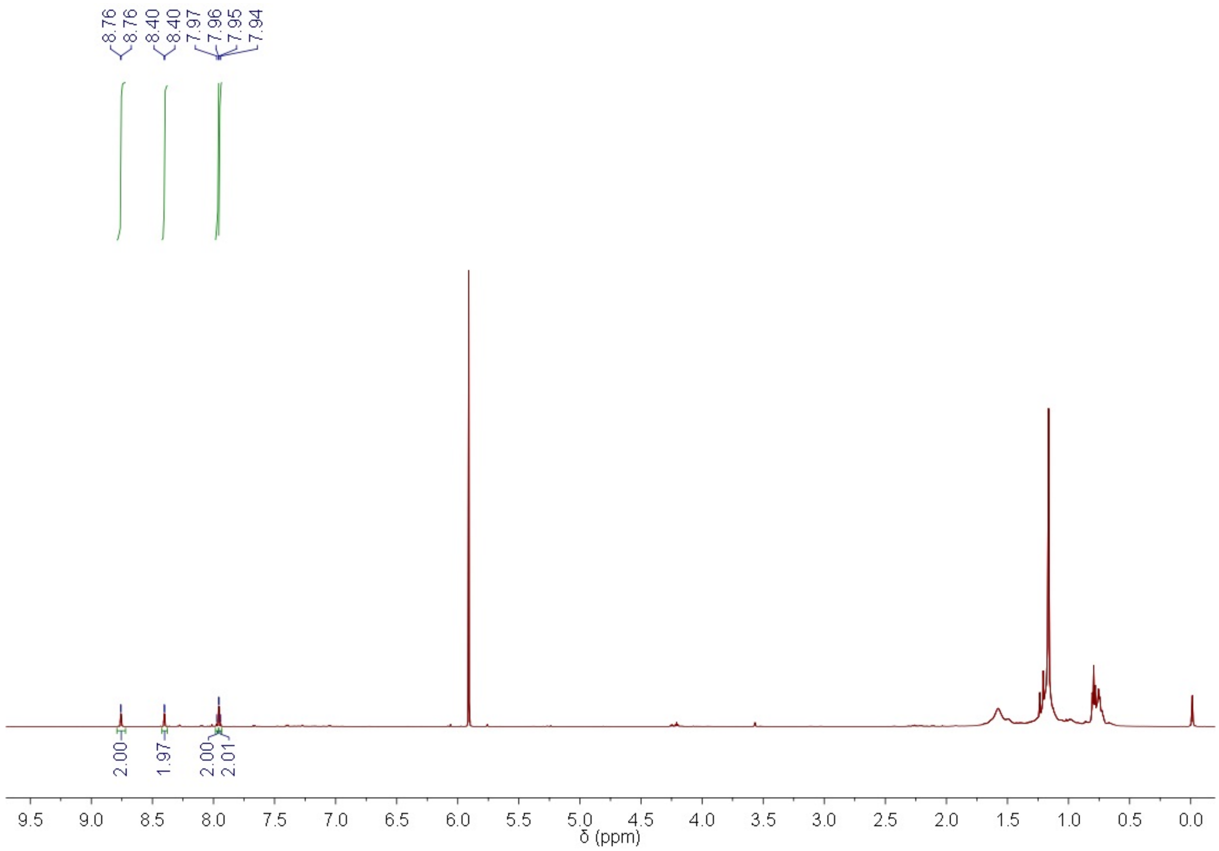

FIG. S13. ^{1}H-NMR spectrum of **1** in $C_2D_2Cl_4$.

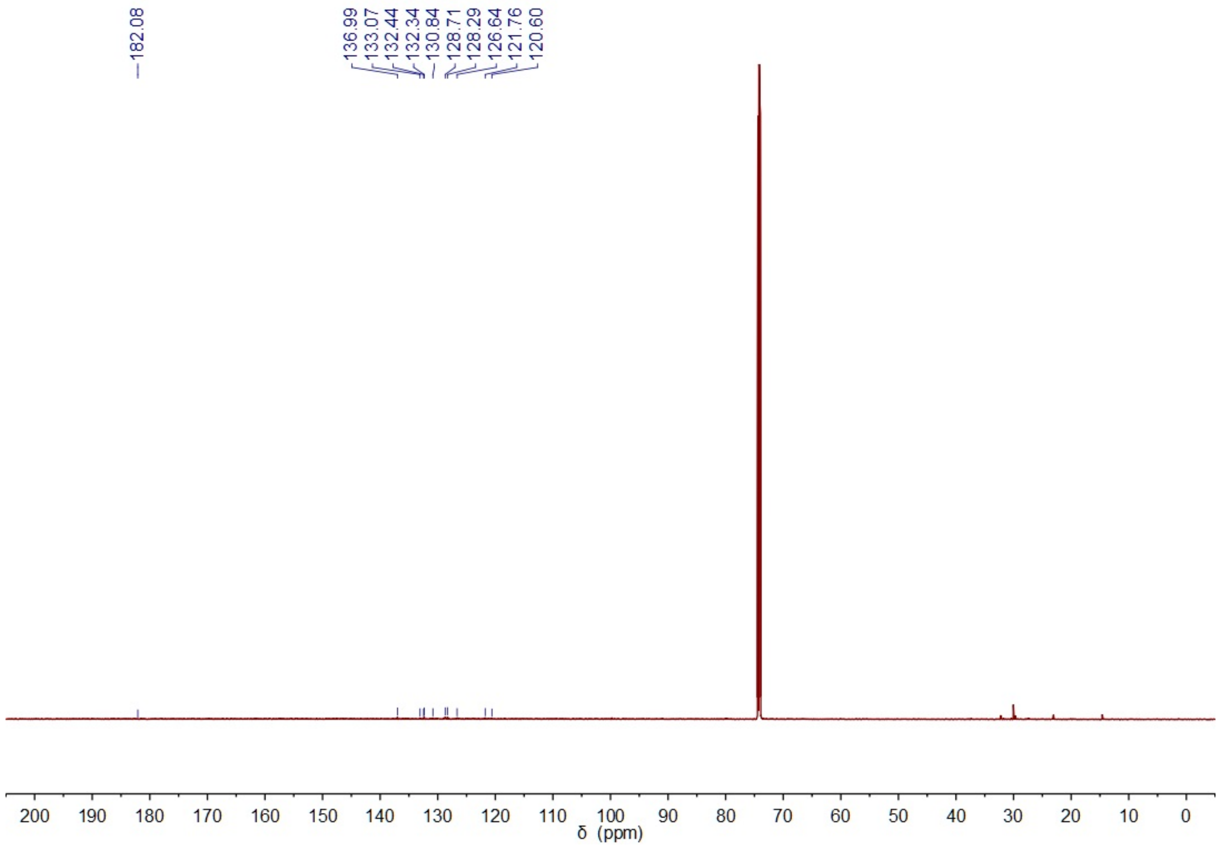

FIG. S14. ^{13}C-NMR spectrum of **1** in $C_2D_2Cl_4$.

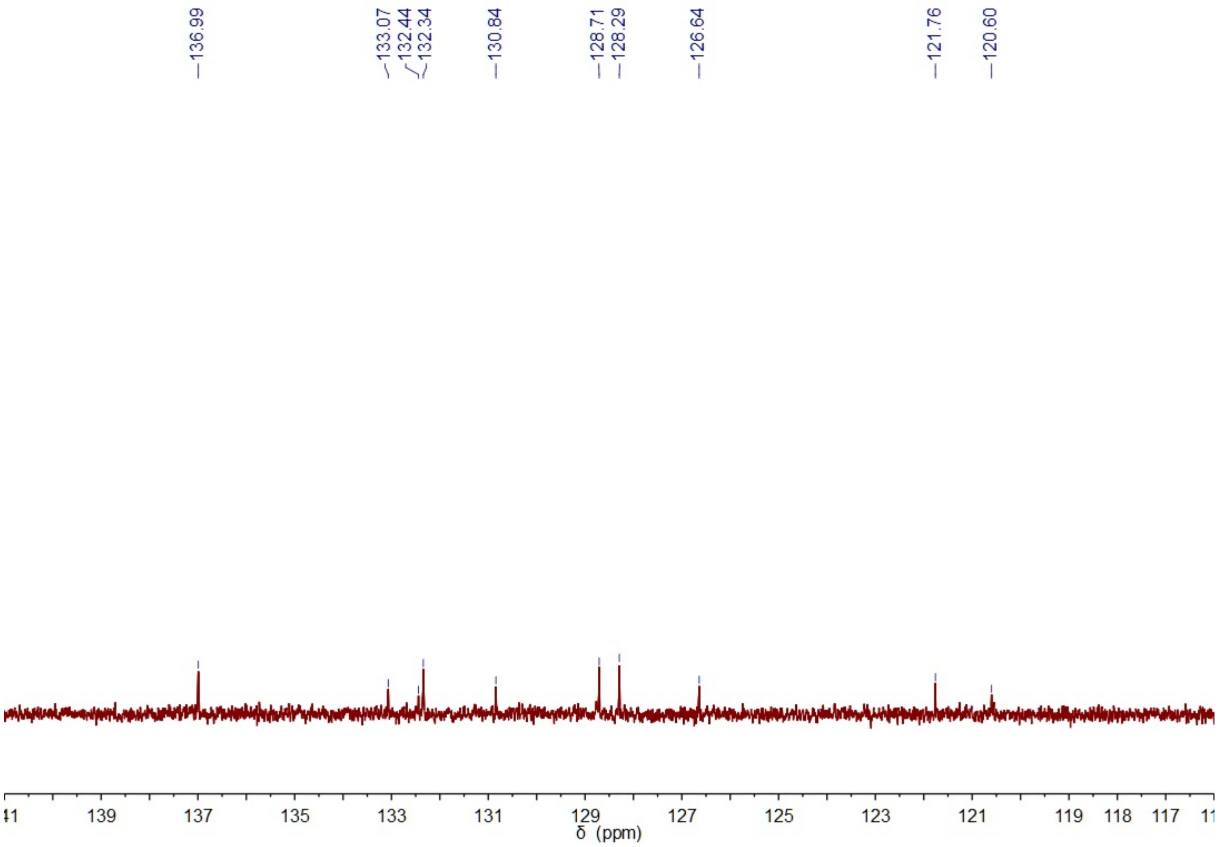

FIG. S15. ^{13}C-NMR spectrum in a narrow range of **1** in $C_2D_2Cl_4$.

[1] Xiang, Qin and Guo, Jing and Xu, Jun and Ding, Shuaishuai and Li, Zhaoyang and Li, Guangwu and Phan, Hoa and Gu, Yanwei and Dang, Yanfeng and Xu, Zhanqiang and Gong, Zongcheng and Hu, Wenping and Zeng, Zebing and Wu, Jishan and Sun, Zhe Stable olympicenyl radicals and their π-dimers. *Journal of the American Chemical Society* **142**, 11022–11031 (2020).

[2] Kulka, Teresa and Panfil, Milosz and Berciu, Mona and Wohlfeld, Krzysztof Nature of spinons in the 1D spin chains. *arXiv preprint arXiv:2303.02276*
